# Slack-based tunable damping leads to a trade-off between robustness and efficiency in legged locomotion

**DOI:** 10.1038/s41598-023-30318-3

**Published:** 2023-02-25

**Authors:** An Mo, Fabio Izzi, Emre Cemal Gönen, Daniel Haeufle, Alexander Badri-Spröwitz

**Affiliations:** 1 Dynamic Locomotion Group, Max Planck Institute for Intelligent Systems, 70569 Stuttgart, Germany; 2 Hertie Institute for Clinical Brain Research and Center for Integrative Neuroscience, University of Tübingen, 72076 Tübingen, Germany; 3 Institute for Modelling and Simulation of Biomechanical Systems, Computational Biophysics and Biorobotics, University of Stuttgart, 70569 Stuttgart, Germany; 4 Department of Mechanical Engineering, KU Leuven, 3001 Leuven, Belgium

**Keywords:** Biomechanics, Mechanical engineering, Musculoskeletal models

## Abstract

Animals run robustly in diverse terrain. This locomotion robustness is puzzling because axon conduction velocity is limited to a few tens of meters per second. If reflex loops deliver sensory information with significant delays, one would expect a destabilizing effect on sensorimotor control. Hence, an alternative explanation describes a hierarchical structure of low-level adaptive mechanics and high-level sensorimotor control to help mitigate the effects of transmission delays. Motivated by the concept of an adaptive mechanism triggering an immediate response, we developed a tunable physical damper system. Our mechanism combines a tendon with adjustable slackness connected to a physical damper. The slack damper allows adjustment of damping force, onset timing, effective stroke, and energy dissipation. We characterize the slack damper mechanism mounted to a legged robot controlled in open-loop mode. The robot hops vertically and planarly over varying terrains and perturbations. During forward hopping, slack-based damping improves faster perturbation recovery (up to 170%) at higher energetic cost (27%). The tunable slack mechanism auto-engages the damper during perturbations, leading to a perturbation-trigger damping, improving robustness at a minimum energetic cost. With the results from the slack damper mechanism, we propose a new functional interpretation of animals’ redundant muscle tendons as tunable dampers.

## Introduction

**Figure 1:**
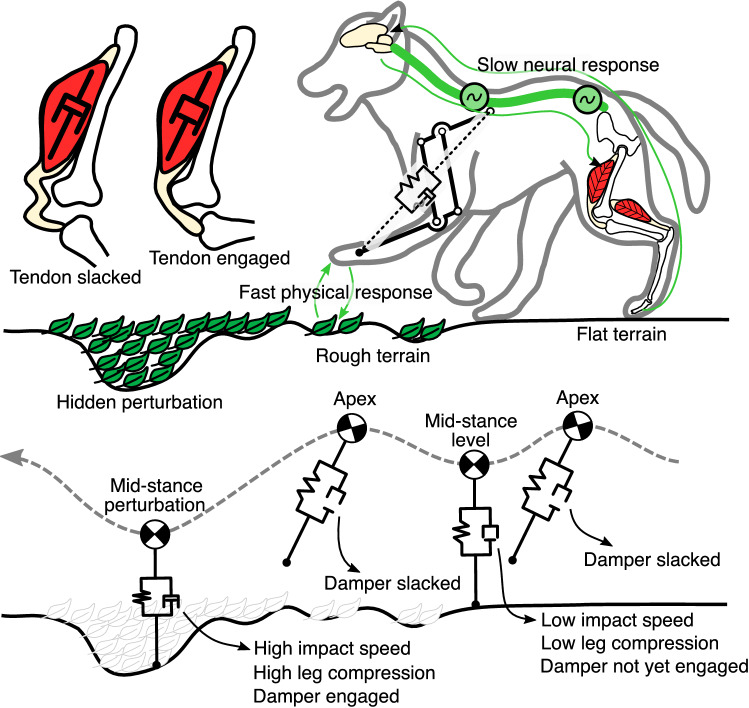
Top: Fast running over ground perturbation is challenging. Due to sensorimotor delays up to 50 ms, the central nervous system struggles to perceive and react to sudden ground disturbances^1^. In contrast, the intrinsic mechanics of the musculoskeletal system act like a spring damper. They produce a physical and, therefore, immediate (< 5 ms) reaction when in contact with the environment. We hypothesize that the leg damping mitigates ground disturbance through adaptive force production and energy dissipation. The tendon’s slack, coupled with the joint’s motion, auto-engages the damper. This creates a trade-off between locomotion robustness and energetic efficiency. Bottom: The damper slack enables perturbation-triggered damping. Sufficiently slacked, the damper does not engage during stance, and only spring-based torque is produced. When encountering a perturbation, the leg’s compression increases further, removing all damper slack, and the damper engages in parallel to the spring.

Animals run dynamically over a wide range of terrain (Fig. 1). The unevenness and changing compliance of natural terrain demand the capability for fast and dynamic adaptation to unexpected ground conditions. However, animals’ neurotransmission delays slow down sensorimotor information propagation^2^, rendering a neuronal response impossible for as much as 5 to 40% of the stance phase duration, depending on the animal size^1^. How animals are able to produce and maintain highly dynamic movements despite delayed sensorimotor information is, therefore, a central question in neuroscience and biorobotics^1,3–5^.

Inherent mechanical properties of muscles facilitate the rejection of unexpected perturbations^6–9^. Muscular tissue possesses nonlinear elastic and viscous-like mechanical properties, which adapt the muscle force instantly to changes in the length or contraction velocity of the muscle-tendon fibers. These mechanical properties enable the neuro-musculoskeletal system to react to external perturbations with zero delay, a capacity termed “preflex”^10,11^.

Intrinsic elasticity and its role in legged locomotion have been studied extensively^12–16^. For instance, tendons, which behave like nonlinear serial springs, store and release mechanical energy during ground contact^12^ and improve shock tolerance^17^. Inspired by this, parallel and series elastic actuators have successfully been implemented in the design of legged robots^18–21^, demonstrating improved robustness at low control effort. In contrast, the functional role that damping plays in legged locomotion is less studied and understood.

Damping can produce a force outcome that is adaptive to the impact velocity. This adaptive force output enhances the effective force output during impacts^22^, minimize control effort^23^, stabilize motion^24–26^ and reject unexpected disturbances^27,28^. Nevertheless, damping is usually minimized in the design of (bio)robotic systems, as it can lead to increased energy consumption. Interestingly, vertebrates seem capable of tuning the damping produced by their muscle fibers^29^. This suggests that tunable damping can be a solution for regulating damping forces and dissipating energy depending on the terrain conditions.

Tunable damping in biorobotics can be implemented through control^30,31^, i.e., virtual damping. Virtual damping poses substantial design constraints. It requires precise velocity estimation, high-frequency control (> 1 kHz), strong actuators to produce sufficient peak forces, and means to dissipate the resulting heat effectively^32–36^. Alternatively, physical dampers can be mounted in parallel to the robot’s joints^37^. A physical damper perceives and responds physically and instantly, requires no controller or computation, shares peak load of actuators, and thus has the potential for fast adaptation to terrain perturbations^38^. Tuning damping with a physical damper mounted to a legged robot proved challenging. Setting a higher damping rate resulted in the expected higher forces, but at reduced leg compression and effective damper stroke^38^. Consequently, the dissipated energy indicated by the work loop area did not increase. Additionally, fix-mounted physical dampers operate continuously and dissipate energy during unperturbed level running. Instead, physical tunable damping should ideally be triggered by the perturbation itself. The damper should engage and self-adjust according to the presence and severity of the ground disturbance experienced during running.

The tendon slack observed in muscle-tendon units^39,40^ and animal-inspired robots^41^ provided us with a design template for implementing tunable damping in a legged system (Fig. 1 Top). Tendon slack length is defined as the “...length beyond which the tendons associated with a muscle begin resisting stretch and producing force”^40^. In other work, the “tendon is strained up to 2%, representing the “stretching out” of the crimped tendon fibrils, before starting to transfer considerable force”^39^. Badri-Spröwitz *et al*. show tendon slack in the flexing motion of the digits of large birds, and implement tendon slack in the related robot^41^. By disengaging the damper from its joint via controlled tendon slack, we expect to adjust the onset, timing, and amount of damper engagement. Moreover, the tendon slack allows for a perturbation-trigger strategy (Fig. 1Bottom). During steady-state running, for example, on flat terrain, the leg compresses without saturating the tendon slack. Once an unperceived ground perturbation increases leg compression further, the tendon displacement will exceed the tendon’s slack and start to auto-engage the damper. This strategy enables adaptive force output triggered by ground perturbations.

We implemented and tested a bio-inspired, physical tunable damping strategy based on tendon slack in this work. We aimed at producing perturbation-triggered damping and improving robustness against ground perturbations. We evaluated this design concept on a robotic leg during vertical and forward hopping, both in steady-state and perturbed conditions. Unlike earlier designs^38^, our slack damper mechanism enabled straightforward adjustment of the damper engagement and energy dissipation. We observed improved hopping robustness due to the adaptive characteristics of our physical damping design, whereas the energetic cost increases. The perturbation-triggered capacity of our slack damper mechanism allows for a more favorable trade-off between robustness and efficiency.

## Results

We designed three experiments to study the proposed design with a hydraulic damper mounted to a robotic leg joint (Table 1). We tested damper slack values of 10, 6, 3, and 0 mm for all conditions. These settings span from full slack (10 mm, minimum effective damping) to no slack (0 mm, maximum effective damping). An open-loop controller produced the robot leg’s locomotion pattern. Without feedback, ground perturbations were invisible to this high-level control (neural circuits), and perturbations could only be compensated by low-level mechanics in the form of a physical response.

We used the vertical hopping setup to investigate the vertical component of locomotion, allowing ground reaction force (GRF) measurement in all steps (Fig. 5e). We introduced step-down perturbation to evaluate the robustness of the system. We used the forward hopping setup, which mounts the leg on a boom structure, to simulate more realistic locomotion dynamics (Fig. 5f). We analyzed forward hopping performance on rough terrain and robustness against ramp-up-step-down perturbation.

All data can be found in Supplementary Table S3–5.

**Table 1:** Experiment design, all experiments are repeated with damper slack values of 10, 6, 3, and 0 mm, from maximum slack to no slack.

Experiment	Terrain	Perturbation Height	No. of perturbation steps	No. of repetitions
Vertical hopping	Step-down	10% LL	1	10
		15% LL		
Forward hopping	Flat terrain	0 mm	15	4
	Rough terrain	${\pm }$5 mm		
	Rough terrain	${\pm }$10 mm		
Forward hopping	Ramp-up-step-down	15% LL	1	10
		30% LL		

### Vertical hopping with step-down perturbation

**Figure 2:**
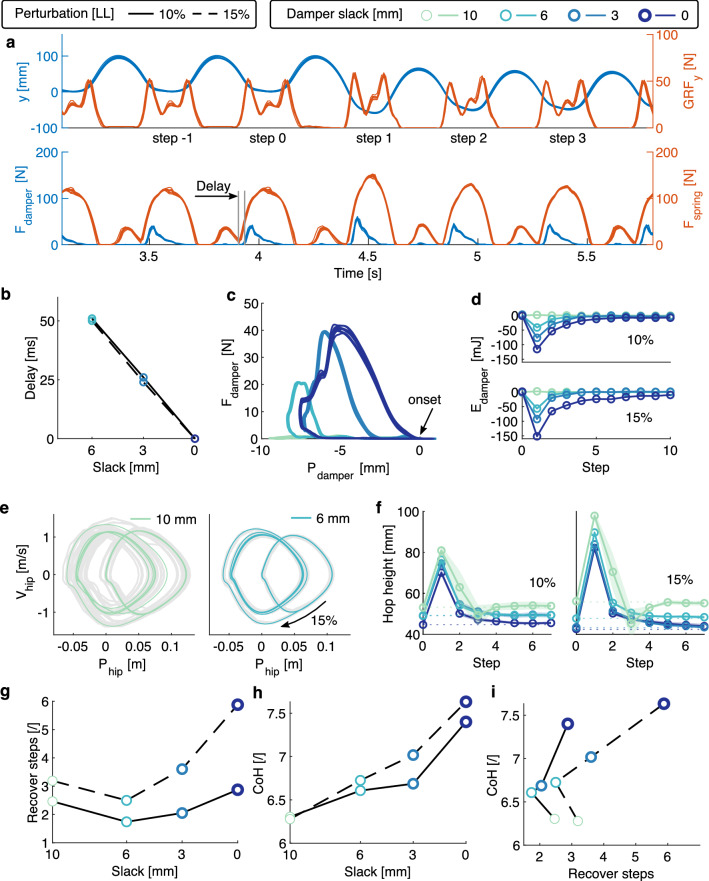
Vertical hopping with step-down perturbation: (**a**) 10-repetition-overlay time-series of hip position *y*, GRF, spring, and damper forces. 15% LL perturbation at step 1 increases the GRF, spring and damper forces due to higher impact speed. The damper starts to produce force with a delay to touchdown due to the 3 mm slack setting. (**b**) This damper engagement delay is adjustable by the damper slack setting. (**c**) The 10-repetition-overlay damper work loop in unperturbed periodic steps shows that the onset position can be reliably tuned and the standby dissipated energy (enclosed area) adjustable. (**d**) The average extra damper dissipated energy during perturbation steps. (**e**) Phase plot of hip position with 10 mm and 6 mm damper slack under 15% LL perturbation. The grey overlay shows the overlap of 10 repetitions of 20 steps, while the darker line is the averaged trajectory. (**f**) The average hopping apex height during perturbation steps. The transparent overlay represents the 95% confidence boundary. (**g**) The relationship between the number of steps to recovery after perturbation and the damper slack settings. (**h**) The relationship between the cost of hopping and the damper slack settings. (**i**) The relationship between the number of steps to recovery to the cost of hopping under different damper slack settings and perturbation levels.

With feed-forward control, the leg hopped in the vertical setup for two perturbation levels and four slack values. Figure 2a shows an example of a time-series of 10 repetitions. The test condition included a perturbation of 15% leg length (LL) and tendon slack of 3 mm (Supplementary Movie S1). At the perturbed step 1, the leg impacted the ground at a higher speed, compressing more. This resulted in higher damper and spring forces than during pre-perturbation levels. We noticed that the damper force did not drop to zero at mid-stance due to the damper’s internal recovery spring.

We found that the tunable slack mechanism was effective in tuning damping. Damper slack adjustments of 0 to 6 mm resulted in a delayed engagement of the damper: from 0 to 50 ms after the onset of the spring force during level hopping (Fig. 2b). The damper’s force-displacement work loops during level hopping confirmed the controllable onset of the damper force (Fig. 2c). The enclosed work loop areas represent the damper’s standby dissipated energy. Damper slack values of 0, 3, 6, and 10 mm can be mapped to standby dissipation of 152, 86, 29, and 1mJ. At the perturbation step, the damper dissipated more energy (65% to 190%) compared to level hopping standby dissipation (Fig. 2d). The extra dissipated energy is associated with the height of the ground drop, showing an adaptive energy dissipation to terrain disturbance. In all tested conditions, the extra dissipated energy converged to 0 in the following steps, indicating recovery to steady-state hopping.

The robustness of the hopping system can be qualitatively assessed by the phase plot of the hip height (Fig. 2e and Supplementary Movie S1). With a 10 mm slack setting, the hopping behavior was the most variable, as shown by the overlay of gray lines, representing 200 steps in 10 repetitions. With a 6 mm slack setting, the phase plot was clean, and the hopping converged to a new ‘limit cycle’ in fewer steps than other settings. A quantitative robustness measurement is the number of steps required to bring the system back to its original hopping height after the perturbation (Fig. 2f). The system’s robustness was highest with the 6 mm slack setting, requiring on average 1.7 and 2.5 steps to recover for 10% and 15% LL perturbation, respectively (Fig. 2g). At stronger perturbations, the robot needed more steps to recover. We measure the energetics of the hopping system by its cost of hopping (CoH, equation (4)). The CoH increased from 6.3 to 7.6 with higher damping or stronger perturbations (Fig. 2h). With a damper slack of 6 mm at 10% LL perturbation, we found 47% faster perturbation recovery in combination with 5% higher CoH compared to 10 mm damper slack (Fig. 2i).

### Forward hopping with continuous perturbation

**Figure 3:**
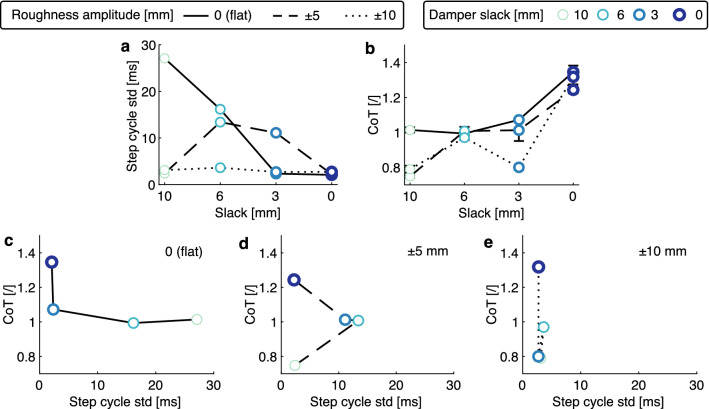
Forward hopping with continuous perturbation: (**a**) The standard deviation of the step cycle time shows that the hopping periodicity is improved with higher damping (less slack). (**b**) The relationship between the CoT and the damper slack settings. (**c**) In flat terrain, the robot’s ability to maintain periodic hopping is improved by higher damping at the cost of CoT. (**d,e**) In the continuous perturbation terrain, high damping is also associated with high CoT and good periodicity.

During forward hopping on the sinusoidal ground, the standard deviation of the step cycle time quantifies the hopping periodicity. In the flat terrain, the standard deviation of the step cycle time decreased from 27 ms to 2 ms with less damper slack, showing improved hopping periodicity with more damping (Fig. 3a). This tendency was less apparent in ±5 and ± 10 mm rough terrain, as the step cycle time variation increased first for the damper slack value 6 mm, then decreased with less damper slack. The energetic cost of forward hopping was measured as the cost of transport^42^ (CoT, equation (5)). The CoT increased from a minimum of 0.75 to 1.35 with increasing damping (Fig. 3b). Both hopping periodicity and CoT were affected by the terrain’s roughness. In flat terrain, increasing damping was associated with improved periodicity and increased CoT (Fig. 3c). At ±5 mm terrain roughness, data for damper slack values of 0, 3, and 6 mm show similar tendency. The 10 mm damper slack shows the best performance with a CoT of 0.75 and a standard deviation of 2 ms cycle time (Fig. 3d). With ±10 mm terrain roughness, the cycle time standard deviation was clustered around 2 mm to 3 mm for all slack settings, while the CoT varied from 0.79 to 1.32. Among these three tested terrains, the strongest damping, i.e., the setting with a slack of 0 mm, showed better periodicity with a cycle time standard deviation of $\approx$2 ms, but with the highest CoT, ranging from 1.24 to 1.35.

### Forward hopping with ramp-up-step-down perturbation

**Figure 4:**
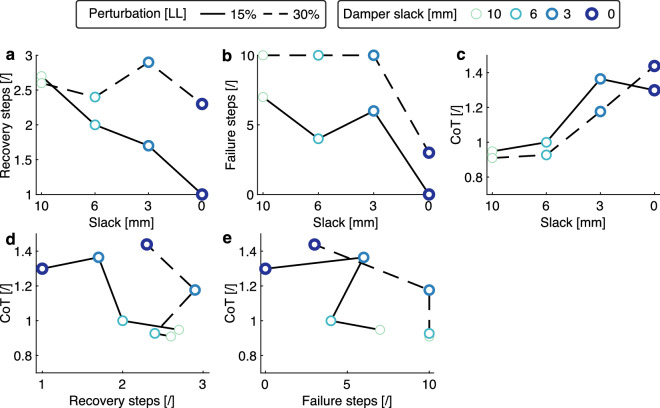
Forward hopping with ramp-up and step-down perturbation: The robustness of the robot system is quantified with the number of steps needed to recover stable hopping (**a**) and the number of failed trials in 10 attempts (**b**). (**c**) The relationship between the CoT and the damper slack settings. (**d**,**e**) show the trade-off between robustness and CoT.

We evaluated the system’s robustness during forward hopping by testing its response to unexpected, sudden perturbations. Thus, we analyzed the robotic leg’s behavior with step-down perturbations in its hopping path. As robustness measurement, we counted the number of steps required for the hopper to recover after the step perturbation. The second measurement of robustness is the number of failures out of ten perturbation attempts. By reducing the damper slack from 10 to 0 mm, the average recovery steps needed by the robotic leg decreased from 2.7 to 1.0 for the 15% LL perturbation and from 2.6 to 2.3 for the 30% LL perturbation (Fig. 4a). Similarly, with more damping, the number of failed trials decreased from 7 to 0 for the 15% LL perturbation and 10 to 3 for the 30% LL perturbation (Fig. 4b). The legged robot was less robust against a stronger perturbation, as it required on average 0.7 more recovery steps or failed, on average, four times more for the two tested perturbation levels. Similar to the other two experiments, the energetic cost of the system increased with more damping, as the CoT increased from 0.95 to 1.44 (Fig. 4c). With a damper slack of 0 mm at 15% LL perturbation, we found 170% faster perturbation recovery in combination with 27% higher CoH compared to 10 mm damper slack (Fig. 4d). With both measurements of robustness, we observed a tendency of increasing robustness at the expense of more energetic cost with higher damping settings (Fig. 4d and e).

## Discussion

The slack damper mechanism allows effective tunable damping. This has three consequences: First, depending on the slack setting, the damper produces an immediate or delayed response to ground impacts (Fig. 2b). Second, the onset of the damper stroke can be reliably set by the tendon slack (Fig. 2c). Third, the mechanical work generated by the damper is tunable, as shown by the change in the size of the enclosed work loops (Fig. 2c). Such a level of tunability of the damper response was not possible in our previous, more canonical approach of controlling the damping rate of the same damper model (implemented in a two-segment leg) via orifice adjustment^38^. In contrast, adjusting the slack of the damper tendon provides an effective strategy for tuning embedded damping in the robotic leg. The slack in the damper tendon system allows the parallel spring to soften the damper impact within tens of milliseconds after the foot touchdown. As a result, the damper produced less force and effective stroke than scenarios with less slack (Eq. (1), Fig. 2c).

In the steps following a sudden drop in ground height, the additional gravitational energy results in 20% to 30% higher touchdown speeds. The damper force and negative work increase accordingly, providing a beneficial mechanical reaction to compensate for the perturbation (Fig. 2d). Therefore, our damper implementation produces mechanical work in an adaptive manner that is consistent with the perturbation level and tunable by just one parameter; the damper tendon slack.

Legged system robustness is required due to the system’s inherent sensor- and control-noise and the imprecision of its motor-control^1,43,44^. Heim *et al.*^45^ quantified task-level stability in a modified spring-loaded inverted pendulum (SLIP) model that includes perturbation-triggered damping, suggesting that increased damping contributes to improved robustness. Legged locomotion simulation studies^24,26^ and muscle experiments^46^ revealed the stabilizing effect of damping. This theoretical evidence motivated our biorobotic setup to explore and characterize damping and its effect on locomotion robustness.

In general, damping improves system robustness. In the vertical hopping experiments, adding a small amount of damping (6 mm slack) led to the fastest recovery from step perturbations (Fig. 2e and g). Above a certain amount of damping, the robotic leg appears to be “over-damped”, as shown by the hopping height over steps. For example, with more damping (slack < 6 mm), the convergence to the pre-perturbation behavior is smoother (Fig. 2f) but requires more steps (Fig. 2g). In forward hopping experiments, more damping improved hopping periodicity (Fig. 3a) and robustness (Fig. 4a and b) without the emergence of an over-damping threshold. Our system performed well in this perturbed condition. It overcame the perturbation 64 times out of 80 trials, despite using the simple feed-forward open-loop controller for forward hopping motion. Although no electronic sensors are utilized to perceive the perturbations, the passive compliance embedded in the leg acts as an intrinsic system of mechanical sensors and actuators, which detect and respond immediately to external disturbances. We believe the adaptive force output from damping plays a key role. A reflex-control mammalian quadruped of similar size to our robot has a total sensorimotor delay of 60 ms^3^. In comparison, the delay of damping force production in the robotic leg is less than 50 ms (Fig. 2b). This confirms that the physical damping force effectively acts faster than reflex control in response to a perturbation.

The improved robustness introduced by the damper system comes at an energetic cost. Higher damping settings (less slack) result in higher energy costs for all the experiments (Figs. 2i, 3b, and 4c). This occurs because the actuators need to produce more power to compensate for the lost energy by damping (Fig. 2c,d) and achieve a steady-state hopping behavior. Tunable damping leads to a trade-off between the robustness and energy cost of the system (Fig. 4d,e). This trade-off implies that hopping can be either robust against perturbations but with a penalty in energy consumption, or be energy efficient but vulnerable to disturbance. Adjusting tendon slack allows for selecting a suitable compromise depending on the terrain.

The benefit of damping for legged systems remains a debate in the field^24,45,47^. Most research on legged locomotion focuses on optimizing a single aspect, such as robustness, stability, or energy consumption. On the contrary, evolution in biology is likely not a single-objective optimization process. Instead, we argue that a more holistic perspective is required to understand the interaction among the many performance metrics characterizing legged locomotion. Therefore, we argue that the locomotion priority can change. As Fig. 1 suggests, less damping is desired to minimize energy consumption during level terrain locomotion. In case of rough terrain, higher damping is preferred to improve the robustness against ground disturbances. Hence, damping is a key to balance the trade-off between robustness and energy consumption.

The advantage of our slack damping mechanism concerning energy consumption is that it allows a perturbation-trigger strategy. The damper tendon slack can be tuned to barely engage at level hopping. It will then engage once a ground perturbation induces higher impact velocities. In this way, the absence of a damper minimizes the dissipating energy during level hopping, while the engagement of the damper improves robustness at ground perturbation steps. This automatic on-off control was impossible with previous damper implementations^48,49^, because damping generated from friction, rheology, eddy currents, and fluid dynamics are hard to switch off completely^37^. Instead of optimizing the adjustment of the nonlinear damping coefficient, our mechanism features a fixed damping coefficient but exploits a slack tendon to create a tunable on-off damping. The proposed slack tendon could also be applied to selectively engage springs. Hence, the tunable tendon slack mechanism offers a new mechanism for adaptive compliant actuator applications.

Besides the adaptive force output of damping, we expect the tunability of damping to provide better hopping behavior, such as transitioning into new terrain. When expecting a more uneven terrain, the damper slack can be adjusted accordingly to gain more robustness against the stronger perturbation. This requires an online slack tuning mechanism and its feedback control strategy. Possibly, a feed-forward controller can be sufficient to produce highly robust running in an uncertain environment^50^. Limited by the hardware implementation, we did not thoroughly investigate an online tuning design. Nevertheless, the four damper slack settings demonstrate the proof-of-concept of online tunable damping.

We consider extending our system with stiffness control in the future. Tunable spring designs have been studied extensively^37^, but a combination with tunable damping is rare. Software online tuning of stiffness and damping has been realized^51,52^, but relies on precise sensing, high-frequency control and strong actuation. Virtual feedback impedance control^53,54^ combined with physical springs and dampers provide software control flexibility and fast physical response^5^. With these improvements, we can readily implement controllers and hardware for versatile and robust locomotion in natural terrains such as gravel.

In summary, this work aims at understanding the tunable damping mechanism in legged locomotion. We proposed the slack damper strategy inspired by muscle tendon slack and tested it in robotic legged hopping. The slack damper mechanism allows effective tunable damping regarding onset timing, engaged stroke, and energy dissipation. This study provides novel insights into the trade-off between energetics and robustness under different damping levels. Additionally, the slack damper design allows for perturbation-trigger damping, resolving the trade-off during locomotion with unexpected perturbation. Our results could inspire future robotic locomotion hardware and controller design.

## Methods

### Biorobotic leg implementation

**Figure 5:**
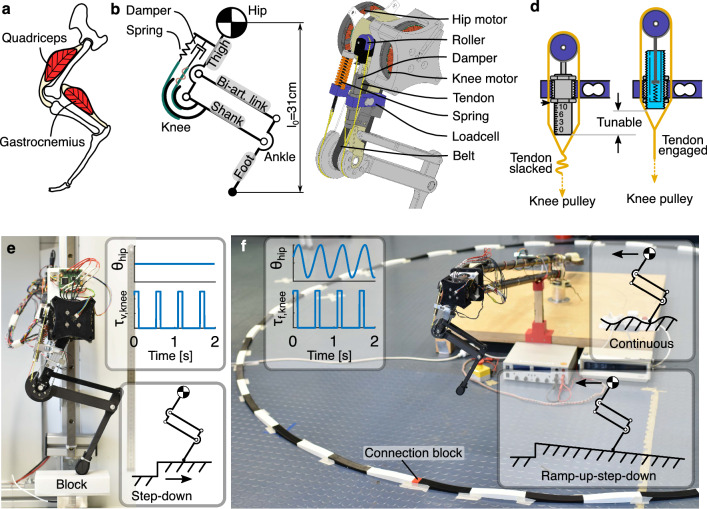
Experiment setup overview. (**a**) Our leg design is inspired by the leg anatomy of mammalian quadrupeds. (**b**) We implement a pantograph leg design with spring and damper representing the passive compliance of the quadriceps and a biarticular segment, simplifying the gastrocnemius muscle and the Achilles tendon. (**c**) The rendering of the leg design shows that the knee joint is coupled to the linear spring, the linear damper through tendons, and the knee motor through a timing belt. (**d**) The slack damper mechanism is realized by the threaded connection between the damper and the loadcell. By rotating the damper, the damper will travel up and down, thus allowing tunable tendon slack. The left schematics illustrates the lowest position of the damper in maximum tendon slack, and the right schematics demonstrates the inner mechanics of the hydraulic damper with minimum tendon slack. (**e**) The vertical hopping setup fixes the robot leg on a vertical slider to test step-down perturbation, which is introduced by removing the perturbation block on top of the force sensor. The top right shows a feed-forward control pattern for hip position and knee torque. (**f**) The forward hopping setup fixes the robot leg on a rotary boom to test continuous perturbation (in photo) and ramp-up-step-down perturbation (Supplementary Movie S3). The top right shows a feed-forward CPG control pattern for hip position and knee torque.

The 3-segment leg design was inspired and simplified from the leg anatomy of small mammalian quadrupeds (Fig. 5a). It consisted of four links forming a pantograph structure (Fig. 5b). A spring and a damper coupled to the knee joint mimicked the passive compliance of the quadriceps muscles. The gastrocnemius muscle and Achilles tendon were simplified as a rigid link to reduce parameter space. The two-degrees-of-freedom leg was fully actuated by two motors (hip and knee). The key design parameters are provided in the supplementary materials (Fig. S1 and Table S1).

The leg was fabricated mostly from off-the-shelf components and 3D-printing (Fig. 5c). The main structural components were 3D-printed using polylactic acid (PLA), except for the foot segment, which was 3D-printed using carbon-fiber-reinforced nylon to improve strength and impact resistance. The hip and knee motors (MN7005-KV115, *T-motor*, 1.3Nm maximum rated torque) were placed co-axially at the hip to reduce leg swing inertia, using a 5:1 planetary gearbox (lgu35-s, *Matex*) to gear them down. The knee torque was transmitted by a timing belt (SYNCHROFLEX 10/T5/390, *Contitech*) with an additional 25:12 gear ratio. We mounted two loadcells (model 3134, *Phidgets*, 20kg) to the spring and the damper to measure forces. The knee spring (SWS14.5-15, *MISUMI*) was designed to hold the leg in stance. Its stiffness of 10.9N/mm was empirically determined to generate three times the body weight of the robot at 10% leg length deflection^55,56^. The knee damper (1210M, *MISUMI*) was selected as the most effective damper from our previous study^38^. Both the spring and the damper were coupled to the knee joint through Dyneema tendons (Climax Combat Speed 250/150, *Ockert*), with a cam radius of 30 mm and 20 mm, respectively. A roller (VMRA20-4, *MISUMI*) was attached to the piston of the damper to transform the tendon tension (“muscle lengthening”) in knee flexion to a push motion on the damper piston. The whole leg weighs 0.94kg, with a resting leg length of 31cm.

### Slack damper mechanism

Tuning an adjustable damper when operating within a legged system is challenging. Higher damping settings make the damper produce larger forces, which in turn can reduce the piston displacement, compromising the projected change in dissipated energy^38^. Therefore, it is difficult to anticipate how adjusting the orifice of the damper internal valve affects the dissipated energy. Instead of regulating the damper’s force by adjusting the orifice size, we propose damping control by adjustment of the damper tendon slack. Tendon slack has been observed in biology, with tendon stretch up to 2% of the nominal tendon length before starting to produce considerable force^39–41^. This is known as the ’toe region’ in the tendon’s stress-strain diagram.

Inspired by this observation, we set a defined tendon slack length when connecting the damper to the knee pulley (Fig. 5d). For our mechanism, the damper body and the loadcell are machined with external and internal threading, respectively. By screwing the damper’s body into the loadcell, we set the damper’s position with a resolution of ±1 mm per turn. The adjustable threading allows for a precise slack control in the range of 0 to 10 mm. Before each experiment, we lock the damper in place with two nuts to prohibit damper body movement.

This slack damper mechanism permitted tunable damping. The damper energy dissipation $E_{damper}$, calculated as the integration of damper force $F_{damper}$ and damper piston displacement *x*, can be controlled by the tendon slack *s* because of two concomitant effects (equation (1)). First, when the ground impact flexes the leg, the parallel spring decelerates leg flexion. At the same time, the tendon slack saturates, thereby softening the engagement conditions for the damper’s piston (more slack *s* $\hat{=}$ less damper force $F_{damper}$). Second, the tendon slack reduces the effective damper piston stroke $\Delta x$ (more slack *s* $\hat{=}$ less piston stroke $\Delta x$). The combination of these two mechanisms—softened (less $F_{damper}$) and delayed (less $\Delta x$) damper engagement—predicts an inverse relationship between the tendon slack *s* and the integrated damper energy dissipation $E_{damper}$.

1 $$\begin{aligned} \left. \begin{array}{ll} E_{damper} = \int F_{damper} \, dx \\ F_{damper} \propto \frac{1}{s}, \; \Delta x \propto \frac{1}{s} \end{array}\right\} \Rightarrow E_{damper} \propto \frac{1}{s} \end{aligned}$$

### Experimental setup

We designed two experimental setups and three perturbation types to evaluate the proposed design in four slack settings.

The vertical hopping setup (Fig. 5e) investigates only the vertical component of locomotion. Such a reduced-order experiment reduced system complexity, allowing ground reaction force (GRF) measurement in all steps. The forward hopping setup (Fig. 5f) fixed the leg on a boom structure, simulating more realistic locomotion dynamics and allowing for more perturbation types.

We focus the investigation on the mechanical response produced by the passive damping embedded in the leg design. Hence, we designed an open-loop locomotion controller such that it could not detect ground perturbation. We tested three types of ground perturbations: step-down perturbation representing a sudden, unexpected disturbance during fast running; continuous perturbation simulating rough terrain conditions, and ramp-up-step-down perturbation combining gradual and sudden disturbance.

We tested damper tendon slack of 10, 6, 3, and 0 mm for each test condition. The damper engaged synchronously with the spring in the 0 mm slack setting. With the 10 mm slack setting, the damper never engaged. Hence, we investigated a wide range of possible slack conditions, from complete to zero tendon slack.

#### Vertical hopping

In the vertical hopping setup (Fig. 5e), the hip of the robot leg was fixed to a vertical rail (SVR-28, *MISUMI*). A force sensor (K3D60a, *ME*) was used to measure the ground reaction force during hopping. The step-down perturbation was realized using a 3D-printed block (PLA) and an automatic block-removal device. The block was placed on top of the force sensor to elevate the ground. Magnets were inserted into the block and the top plate of the force sensor to prevent relative sliding during the leg impact. The block-removal device was a lever arm actuated by a servo motor (1235M, *Power HD*). The arm pushed away the block during the aerial phase of a hopping cycle (Supplementary Movie S1). This automatic block-removal device was needed to remove the perturbation block within the aerial hopping phase reliably (200 ms in our experiments).

The vertical hopping setup was instrumented as follows. The hip position was measured by a linear encoder (AS5311, *AMS*). The loadcells (spring and damper) and the ground reaction force sensor readings were amplified (9326, *Burster*) and then recorded by a microcontroller (Due, *Arduino*) with internal 12-bit ADC. The motor position was measured by a 12-bit rotary encoder (AEAT8800-Q24, *Broadcom*). We used an open-source motor driver (Micro-Driver^36^) for motor control, current sensing, and encoder reading, which runs dual motor field-oriented control at 10 kHz. We monitored the motor driver current with a current sensor (ACS723T-AB, *Allegro Microsystems*). A second microcontroller (Uno, *Arduino*) was implemented to control the servo motor for automatic block removal. A single-board computer (Raspberry Pi 4B) was used to centralize and synchronize all sensor readings and motor commands in 1 kHz.

We implemented a Raibert-like^57^ open-loop controller for vertical hopping. The hip was position controlled with a PD controller to keep a vertical posture. The knee was torque controlled to produce a defined torque at a given duty cycle, typically during the second half of the stance phase. Motor commands are illustrated in the inserted plots in Fig. 5e. Control parameters for a stable hopping gait were found through manual tuning, resulting in a 450 ms cycle time with 100 ms knee motor push-off. The knee torque was tuned for each setting of the damper tendon slack to maintain the same hopping heights across tested conditions (Supplementary Table S2).

We tested two perturbation levels: 31 mm and 47 mm, equivalent to 10% and 15% of the leg length, respectively. For each hopping trial, the robot hopped for 1min. We analyzed ten steps before and after the perturbation. Each hopping condition was repeated ten times. We recorded in total 80 trials; two perturbations $\times$ four slack settings $\times$ ten repetitions.

#### Forward hopping

In the forward hopping setup (Fig. 5f), the robot leg was mounted on a boom in a four-bar design. This mount permits only horizontal and vertical motion in the robot’s sagittal plane. The length of the boom was 1.613m, and the travel distance of a complete revolution was around 10m. The boom design is openly available^58^.

The instrumentation of the forward hopping setup was similar to that of the vertical hopping setup. The force measurement and the automatic block-removal device were incompatible with the boom setup and were removed. All the other sensors remained. Horizontal and vertical motions of the rotating boom were measured by two 11-bit rotary encoders (102-V, *AMS*).

We generated the forward motion of the robot leg using a feed-forward central pattern generator (CPG). In most vertebrates, CPGs contribute to controlling rhythmic motion^59^, such as locomotion. We implemented a CPG controller for the hip angle trajectory $\theta _{hip}$:

2 $$\begin{aligned}{} & {} \theta _{hip}=A_{hip} \cos (\Phi )+O_{hip} \end{aligned}$$

3 $$\begin{aligned}{} & {} \Phi = {\left\{ \begin{array}{ll} \frac{\phi }{2D} &{} \phi <2\pi D_{vir}\\ \frac{\phi +2\pi (1-2D_{vir})}{2(1-D_{vir})} &{} \text {else} \end{array}\right. } \end{aligned}$$

where $A_{hip}$ is the hip angle amplitude, $\Phi$ the hip angle phase, $O_{hip}$ the hip angle offset, $D_{vir}$ the virtual duty factor as the fraction of time when the leg moves forward, and $\phi$ the oscillator’s linearly progressing phase. The knee motor was torque controlled to generate push-off force in the late stance, following a fixed square-wave pattern as in the vertical hopping with the same frequency as the hip CPG. The motor commands are shown in the overlay plots of Fig. 5f. For ease of comparison, the control parameters (Supplementary Table S2) remained the same for all forward-hopping experiments.

To replicate rough terrain in a controlled way, we designed 3D-print tracks with a sinusoidal profile (Fig. 5f). The circular track was built from 3D-printed blocks. These were serially connected and taped to the floor. Each block is 360 mm long, and 27 blocks fit the circumference of the hopping path. A single, shorter connection block was added (red, Fig. 5f). This connection block prevents the hopping cycle from being entrained by the terrain harmonic perturbation pattern, e.g., repeatedly stepping onto the exact position of a cycle length of the track. We tested two rough terrains, with the amplitude of the sinusoidal perturbation being 5 mm and 10 mm. In addition, we also tested hopping on flat terrain. For each trial, the robot performed a total of six revolutions. We cropped the first and the last revolution from the recorded data and analyzed the remaining four revolutions (60 steps per condition).

Further, we designed ramp-up-step-down perturbations to disturb stable hopping during forward locomotion. Within a revolution’s 10m hopping path, we built a slope of 3m length for the robot leg to gradually climb and jump off. We tested two perturbation heights: 47 mm and 93 mm, equivalent to 15% and 30% of leg length, respectively. For each trial, the robot leg performed 12 revolutions. We cropped the first and the last revolution from the recorded data and analyzed the remaining ten revolutions (150 steps per condition).

#### Data analysis

The ground reaction force and vertical position data were filtered for the vertical hopping experiments with a 4th-order zero-lag Butterworth filter. The loadcells were calibrated to output force reading only at leg flexion. The spring and damper force data were smoothed using a moving average filter with a filter span of 5 samples. The boom encoder data were filtered with a 4th-order zero-lag Butterworth filter for the forward hopping experiments. The cutoff frequencies (9–19 Hz) of the Butterworth filter were determined by residual analysis^60^.

The recovery steps in the vertical hopping experiment were calculated by first computing the average hop height before perturbation as a reference height (dotted lines in Fig. 2f) and then finding the post-perturbation hop height that intersected with the $\pm 4\%$ boundary of the reference height^21^. The cost of hopping was calculated by normalizing the electric energy consumption $E_{elec}$ of one hopping step to the system’s gravitational potential energy at the apex.

4 $$\begin{aligned} CoH = \frac{E_{elec}}{m \cdot g \cdot h_{apex}} \end{aligned}$$

where *m* is the robot mass, *g* the gravitational acceleration, $h_{apex}$ the apex height position.

We defined two measurements for evaluating the robustness of forward hopping after the ramp-up-step-down perturbation. The recovery steps were defined as the number of steps needed by the robot leg to recover its stable hopping after the step-down perturbation. This metric quantified how fast the robot system can recover from perturbation, and it was measured by visual inspection of the video recordings and kinematic data. The failure step metric quantified the number of failures after a perturbation was applied. We identified two failure modes from the video recordings: the robot leg could slip or stop after the perturbation (Supplementary Movie S3). The number of failures was visually counted from the video recordings. The CoT was calculated by the electric energy consumption per distance traveled *d*, normalized by the robot weight.

5 $$\begin{aligned} CoT = \frac{E_{elec}}{m \cdot g \cdot d} \end{aligned}$$

All data were processed with Matlab (R2021b, *MathWorks*).

## Supplementary Information

Supplementary Information 1.

Supplementary Information 2.

Supplementary Information 3.

Supplementary Information 4.

## Data Availability

All data needed to evaluate the conclusions of the paper are available in the paper or the Supplementary Information. Additional data and the computer-aided design model of the robot leg are available from https://doi.org/10.1038/s41598-023-30318-3.
