# Supplementary material for: Slack-based tunable damping leads to a trade-off between robustness and efficiency in legged locomotion
Source: Sci Rep. 2023 Feb 25;13:3290. doi: 10.1038/s41598-023-30318-3 (PMC9968281; doi:10.1038/s41598-023-30318-3)
Supplement: Supplementary file 1 — Supplementary Information 1. [file 41598_2023_30318_MOESM1_ESM.pdf]

Supplementary Materials for

Slack-based tunable damping leads to a trade-off between robustness and efficiency in legged locomotion

An Mo[1,*], Fabio Izzi[1,2], Emre Cemal Gönen[1], Daniel Haeufle[2,3], and Alexander Badri-Spröwitz[1,4]

[1]Dynamic Locomotion Group, Max Planck Institute for Intelligent Systems, Stuttgart, 70569, Germany
[2]Hertie Institute for Clinical Brain Research and Center for Integrative Neuroscience, University of Tübingen, Tübingen, 72076, Germany
[3]Institute for Modelling and Simulation of Biomechanical Systems, Computational Biophysics and Biorobotics, University of Stuttgart, Stuttgart, 70569, Germany
[4]Department of Mechanical Engineering, KU Leuven, Leuven, 3001, Belgium
[*]Corresponding author: An Mo, mo@is.mpg.de

The PDF file includes:

Figure S1
Tables S1 to S5
Legends for movies S1 to S3

Other Supplementary Material for this manuscript includes the following:

Movies S1 to S3: https://youtu.be/Sa-q-5NucGY
CAD model of the robot leg and data analysis: https://doi.org/10.17617/3.THJWG8

1 Leg design parameters

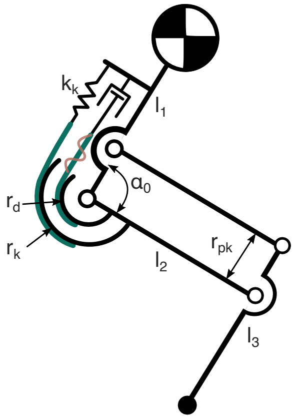

Figure S1. Schematics of the leg with the key design parameters.

**Parameters**		**Value**
Robot mass - vertical hopping	m_v	1.94 kg
Robot mass - forward hopping	m_f	0.94 kg
Leg resting length	l_0	310 mm
Segment 1 length	l_1	150 mm
Segment 2 length	l_2	150 mm
Segment 3 length	l_3	150 mm
Knee spring pulley radius	r_k	30 mm
Knee damper pulley radius	r_d	20 mm
Knee spring stiffness	k_k	10.9 N/mm
Bi-articular insertion radius	r_{pk}	32 mm
Knee resting angle	α_0	100°

Table S1. Robot design parameters.

2 Robot control parameters

Parameters		Value
**Vertical hopping**		
Hopping frequency	f_v	2.2 Hz
Knee torque amplitude	τ_v	4.0 - 4.3Nm
Knee duty cycle	–	0.22
**Forward hopping**		
Hip amplitude	θ_{hip}	18°
Hip offset	O_{hip}	2°
Hopping frequency	f_f	1.85 Hz
Hip virtual duty factor	D_{vir}	0.4
Knee torque amplitude	τ_f	1.3 Nm
Knee phase shift	–	0.75
Knee duty cycle	–	0.2

Table S2. Robot control parameters

3 Experimental results

Perturbation [LL]	Damper slack [mm]	Hop height [mm]	CoH [/]	Recovery steps [/]	Delay [ms]	E_d [mJ]
10%	10	53.3	6.3	2.5	-	1
10%	6	49.3	6.6	1.7	51	26
10%	3	49.1	6.7	2.0	26	117
10%	0	44.7	7.4	2.9	0	186
15%	10	55.8	6.3	3.2	-	1
15%	6	47.8	6.7	2.5	50	29
15%	3	43.2	7.0	3.6	24	86
15%	0	42.4	7.6	5.9	0	152

Table S3. Experimental results of vertical hopping with step-down perturbation. The energy dissipated by the damper (E_d) is calculated by integrating the damping force with respect to the damper compression (Fig. 2c).

Roughness amplitude [mm]	Damper slack [mm]	Speed [m/s]	CoT [/]	Step cycle std [ms]
0	10	0.80	1.01	27.1
0	6	0.79	0.99	16.2
0	3	0.71	1.07	2.4
0	0	0.67	1.35	2.1
±5	10	0.76	0.75	2.4
±5	6	0.76	1.01	13.4
±5	3	0.74	1.01	11.1
±5	0	0.68	1.24	2.2
±10	10	0.76	0.79	3.1
±10	6	0.71	0.97	3.6
±10	3	0.72	0.80	2.7
±10	0	0.66	1.32	2.7

Table S4. Experimental results of forward hopping with continuous perturbation.

Perturbation [LL]	Damper slack [mm]	Speed [m/s]	CoT [/]	Recovery steps [/]	Failure steps [/]
15%	10	0.81	0.95	2.7	7
15%	6	0.78	1.00	2.0	4
15%	3	0.72	1.36	1.7	6
15%	0	0.68	1.30	1.0	0
30%	10	0.80	0.91	2.6	10
30%	6	0.75	0.93	2.4	10
30%	3	0.73	1.18	2.9	10
30%	0	0.64	1.44	2.3	3

Table S5. Experimental results of forward hopping with ramp-up-step-down perturbation.

4 Supplementary Videos

Movie-S1: Vertical hopping with step-down perturbation. The leg is hopping on a block whose height is 15% of the leg length. The slack of the damper is set to 3 mm. The first part of the video shows the experiment in real-time. In the second part, slow motion of the same experiment is repeated. In both cases, hip position y, GRF, spring and damper forces are plotted synchronized to the video. In the last part, the phase plots of the all experiments show the relation between hopping speed and the hopping position.

Movie-S2: Forward hopping with continuous perturbation. The leg moves forward by hopping on the sinusoidal terrain with ± 10 mm amplitude. The damper is fully engaged, i.e., the slackness is 0 mm. After the leg completes one full rotation on the terrain, the video shows frames taken by the high-speed video camera.

Movie-S3: Failure modes of forward hopping with ramp-up-step-down perturbation. The leg moves on the flat surface, and it gradually climbs on the ramp to jump off. The perturbation height is 30% of the leg length, and the damper slack is set to 3 mm. The bottom-right plot shows the synchronized hip position in planer motion. The video shows three cases: slipping, stopping, and the good response after the step-down perturbations. The slipping case can be identified by audio irregularity.
